# Intelligence, Correspondence, &c.

**Published:** 1835-01-01

**Authors:** 


### INTELLIGENCE, CORRESPONDENCE, &c.

We have received a letter from Guernsey, impugning certain statements made by Dr. Hoskins, and copied by us from the Lancet of July 29th last. The passage will be found in page 487 of our October number. The letter contains the following averments :—

" I was sorry to see an extract from page 814 of the Lancet for the 29th of August last, copied into the last number of your Journal, purporting to be a clinical lecture of Mr. Wardrop's, in which he quotes Dr. Hoskins' extraordinary successful treatment of cholera in the Guernsey Hospital by solutions of salt. Now, Sir, from what this gentleman's conviction of the superior efficacy of that remedy arose I cannot divine, but positively assert that he had nothing directly or indirectly to do with the Cholera Hospital, and that *no cases were ever treated there* by *salt and water alone.* I have often observed that numerical details are brought forward in support of fictitious cases casting a shade of reality over them. If Dr. H. has at any time stood by the bed-side of 62 cholera patients in *the third stage* of *cholera*, and *cured* or *witnessed the cure* of 46 *of them, by salt and water alone,* I fearlessly assure you that such cures with such means were never made in this island,—in support of what I here advance, I beg to refer you to the members of the late Board of Health:—my only object in trespassing on your valuable time is to prevent any of your numerous readers from being misled by such egregious perversion of facts as the extract from Dr. H.'s letter contains. I have the honor to be, Sir, your humble servant, ————."

It is incumbent on Dr. H., we think, to adduce testimonies from eye-witnesses to rebut this statement.

The review of Andral's work has been received, but we are unable to appropriate it, as the work itself was previously in the hands of one of our own reviewers. We have lost the address of the writer ; but the paper is left with our publisher, 32, Fleet Street.

### TIEDEMANN'S PHYSIOLOGY.

We have received the first volume of Tiedemann's " Systematic Treatise on Comparative Physiology introductory to the Physiology of Man," translated from the German by Drs. Gully and Hunter Lane ; and we have no hesitation in saying that it is one of the most interesting volumes which we have ever perused. Although it is the *first* volume, and the only one yet published by the distinguished author himself, it forms, nevertheless, a complete system of *comparative* physiology in itself—being introductory to human physiology, which may or may not follow from the same pen. The work is quite incapable of analysis, but we recommend it to the perusal of our readers in the most strenuous and sincere manner.

In the press and speedily will be published, and (by permission) dedicated to Sir Henry Halford, Bart., &c. &c. &c. " A Practical Compendium of the Diseases of the Skin, including a particular Consideration of the most intractable and of those considered Syphilitic ;" by Jonathan Green, M.D., Member of the Royal College of Surgeons, and formerly Surgeon in His Majesty's Royal Navy.

Dr. Bostock's Plan of Medical Reform has come to hand ; but as it has been published in the weekly periodicals, we have delayed its insertion for the present. The tumult of political affairs, and the struggles between conservatives and reformers, will, we fear, arrest for a time the progress of medical reform. We shall wait and watch.

Many other Notices and Answers to Queries we are obliged to omit till next Number.

N.B. The Index (price 3*s*.) to the first 20 Volumes of the New Series is published. Only a limited Number were printed, and they will soon be out of print.

